# Maternal mortality in Mexico, beyond millennial development objectives: An age-period-cohort model

**DOI:** 10.1371/journal.pone.0194607

**Published:** 2018-03-21

**Authors:** Román Rodríguez-Aguilar

**Affiliations:** Engineering Faculty, Anahuac University, Estado de Mexico, Mexico; National Institute of Health, ITALY

## Abstract

The maternal mortality situation is analyzed in México as an indicator that reflects the social development level of the country and was one of the millennial development objectives. The effect of a maternal death in the related social group has multiplier effects, since it involves family dislocation, economic impact and disruption of the orphans' normal social development. Two perspectives that causes of maternal mortality were analyzed, on one hand, their relationship with social determinants and on the other, factors directly related to the health system. Evidence shows that comparing populations based on group of selected variables according to social conditions and health care access, statistically significant differences prevail according to education and marginalization levels, and access to medical care. In addition, the Age-Period-Cohort model raised, shows significant progress in terms of a downward trend in maternal mortality in a generational level. Those women born before 1980 had a greater probability of maternal death in relation to recent generations, which is a reflection of the improvement in social determinants and in the Health System. The age effect shows a problem in maternal mortality in women under 15 years old, so teen pregnancy is a priority in health and must be addressed in short term. There is no clear evidence of a period effect.

## Introduction

Maternal health is an indicator of development and inequality levels of a nation, for being a reflection of poverty and social exclusion. The effect of maternal death in the related social group has multiplier effects, since it involves in most cases family dislocation, economic impact (sometimes women provide their households) and the interruption of the normal social development of orphans. According to data from the World Health Organization, in 2015 303,000 women died due to complications during pregnancy or birth; 99% corresponded to developing countries and most deaths could have been avoided. Motherless children have from 3 to 10 more probabilities of dying in the two following years of the mother's death.

In Mexico, this has been in the agenda of health policy; various plans and programs have been implemented to reduce maternal mortality, since a few decades ago. The maternal mortality indicator was part of the Millennium Development Goals (MDGs), the goal for 2015 was a rate of 22.3 maternal deaths per every 100,000 live births. The indicator goal is not achieved yet, but significant progress has been made, in 24 years, from 1990 to 2014, the maternal mortality ratio (MMR) has decreased 56%, which means an advance of 75% in the fulfilment of the MDGs target of reaching 22.2 in 2015.

Between 1990 and 2015, the world MMR (the number of maternal deaths per each 100,000 live births) was just reduced in 2.3% per year. In some countries, the annual reductions of maternal mortality between the year 2000 and 2010 overcame the necessary 5.5% to reach the MDGs. For these reason the countries have adopted a new goal to reduce even more maternal mortality. Then maternal mortality ratio was established as one of the goals of Sustainable Development Goals to reduce the global MMR to less than 70 per 100,000 live births and to ensure that no country has a maternal mortality rate that exceeds the double of the world average [1].

There is a large production of research related to maternal mortality, but from the clinical point of view, there is little literature focused on the evaluation and analysis of the policies implemented to attack the problem of maternal mortality. In the case of Mexico, focused work has been done in some states, [2] analyzes the main causes of mortality in Mexicali Baja California Mexico, by conducting an epidemiological, cross-sectional and prospective study. The main results show a higher frequency of maternal mortality was in young women, 70% without prenatal control. Hemorrhage secondary to ectopic pregnancy was the main cause of death. [3] carried out a meta-analysis about results and challenges in maternal health in Latin America. The main findings are that region’s performance was below the global average and short of the 75% reduction set in Millennium Development Goal 5 for 2015. The main outcomes show that research on maternal health in the countries where the most impoverished populations of the world are living is not always aligned with their compelling needs. [4] analyze observed results in maternal health for medium and low-income countries focusing the analysis on the causes of maternal death related to the performance of the health system, the meta-analysis carried out considers health interventions as the strengthening of the systems, health promotion and clinical interventions. Was identify several mismatches were noted between research publications, and the burden and causes of maternal deaths.

Other studies conducted in Mexico focus on the detection of risk factors related to maternal mortality, as is the case of the study conducted by [5] for a cohort of 550 women in an obstetrics and gynecology hospital in Guanajuato Mexico. Some significant socioeconomic, medical, and obstetric risk factors of maternal mortality in Mexico have been identified. Such risk factors could play an important role in identifying women who are at highest risk of maternal mortality.

[6] analyzes the trends of maternal mortality in Mexico and the differences between states, with the objective of identifying determinants of maternal mortality, as well as analyzing some of the programs implemented in Mexico to reduce mortality maternal from 1990 to 2010 period. The main findings show a declining trend in MMR over this period, the actual decline has been slower than the expected one. The study finds that the use of contraceptive methods has a negative and significant relationship with the MMR that supports other studies findings that access to the means of planning childbearing is associated with lower levels of maternal mortality.

[7] analyze empirical data on maternal deaths that occurred between 2010 and 2013 in Mexico using statistical models (negative binomial regression, survival analysis and multiple cause analysis), linking databases of the Deliberate Search and Reclassification of Maternal Deaths (BIRMM) and the Birth Information Subsystem (SINAC) of the Ministry of Health throw an analysis of the distribution of time after delivery of maternal deaths. The main results show the reported MMR decreased by 5% per year between 2010 and 2013, the MMR due to late and sequelae-related deaths doubled from 3.5 to 7 per 100,000 live-births in 2013. The multiple cause analysis showed a strong association between the excluded deaths and obstetric causes. It is suggested to review the construction of the MMR by including all deaths due to pregnancy and childbirth into the Maternal death definition.

In this work maternal mortality was analyzed based on two main visions that try to explain the causes of maternal mortality: a) from the perspective of social determinants and b) those attributable to deficiencies in the Health System. Hitherto, there is no conclusive evidence in terms of which is the leading cause of maternal death; in a multicultural country and with a major geographic dispersion, such as Mexico, maternal mortality remains a multifactorial problem, which is partly explained by the social determinants and also by failures of the Health System.

In Mexico comparing statistically the average observed deaths in the period 2002–2014 through the mean difference interesting results were found, because contrary to what was expected it is noted that there are no statistically significant differences between the average deaths of mothers with and without health affiliation. For their part, the educational and marginalization levels, and receiving or not medical care during childbirth did show statistically significant differences in average deaths. According to what was expected, the influence of variables related to social conditions and to the health system that impact maternal mortality was identified.

The programs implemented to date in Mexico have had positive effects but have not been sufficient to reduce maternal mortality, especially if the indicators of maternal mortality in vulnerable population are analyzed. In Mexico, 46.2% of its population live in poverty and 9.5% in extreme poverty, a component that defines this poverty level is the lack of access to health services [8]. This population is the one that shows higher levels in maternal mortality indicators, there is currently no program focused on reducing maternal mortality in vulnerable population.

In order to analyze the impact of transformation of the Health System in the past years through the period effect, as well as the age effect and the effect on the cohort when determining maternal mortality in Mexico; was estimated an Age-Period-Cohort model (APC) using information of Ministry of Health for the period 2002–2014. APC models are a widely used tool in research related to social epidemiology, because it allows to identify in isolation the effects of age, period and cohort when determining mortality rates or incidences. Therefore, they represent a useful tool for the analysis of health care.

The work is structured as follows: in the first section, the materials and methods are analyzed, then the main results are showed and finally, discussion and recommendations of public policy to reduce maternal mortality in Mexico.

## Materials and methods

Information from the Ministry of Health and the National Institute of Geography and Statistics corresponding to the statistics generated on maternal mortality for the period 2002–2014 were used, whose information includes a set of variables related with maternal deaths in Mexico, as socioeconomic conditions of women and another related to the process of medical care they received during pregnancy and at the time of death (S1 Dataset).

The paper addresses the problem of maternal mortality from two perspectives, from the social determinants and from the health system point of view. Based on this approach, a public policy analysis is carried out in the first section, documenting the main causes of maternal mortality in Mexico, as well as a brief description of the plans and programs implemented in Mexico to reduce maternal mortality.

Was released an analysis of mean differences to identify the influence of variables related to social determinants and to the health system in maternal mortality. Finally, to give greater robustness to the analysis we propose an Age-Period-Cohort model.

### Age-period-cohort model

The APC model permit analyze the evolution of maternal mortality in Mexico and its relation with three key variables, the age of the mother, birth cohort and death period. Allow identify causal relationships between these variables and the MMR. Based on information from de Ministry of Health and the *Instituto Nacional de Estadística y Geografía (INEGI)* about maternal mortality, a data base was built per age, date of dead and cohort for the 2002–2014 period (historical information available). In addition, information about registered births in Mexico through the Birth Information Subsystem of the Ministry of Health for the same period was used. The model seeks through a robust method assess evolution of maternal mortality in Mexico in terms of the axes of analysis of this document: (a) social determinants and (b) Health Care System.

Generalized linear model of Poisson was estimated (Eq 1), considering maternal mortality (women-year), age, year of death and birth cohort. Taking as dependent variable, number of deaths per age (*i*), period (*j*) and cohort(*k*). It is assumed that maternal deaths are distributed as a Poisson distribution function with average *θ_ijk_*. The independent variables analyzed were age, year of death and birth cohort. However, estimating a model of these features there are specification problems due to the related variables, since the cohort is determined as the difference between period and age. To solve the problem there are various methods, [9] use penalty functions, [10] use penalty functions estimating the models through Poisson regressions. [11,12] uses the method of estimate functions limiting the analysis to effects that remain constant with any of the models of three factors. This work uses the proposal raised by [13] employing independent variables as continuous applying restricted cubic splines (S1 Table).

$$log\left\{\frac{\theta_{ijk}}{N_{ijk}}\right\}=\mu +\alpha_{i}+\beta_{j}+\gamma_{k}$$ (1)

The APC models are interpreted according the assessed effect; in case of age, the results refer to mortality rates (or incidence) per each 100,000 people. For period and cohort purpose, the results are presented in a semi-logarithmic scale and can be interpreted as relative risks (rate ratio). Poisson models assume that the number of observed deaths follows a Poisson distribution by expressing mortality rate depending on variables like sex, age and period of death, which in turn allow to build the birth cohort. The objective is to identify the effect of these variables separately in the mortality rate and incidence. This implies that the variation of mortality rate can be attributed to three main causes:

1. Age: represents changes in mortality rate due to the person's age, regardless the birth cohort.
2. Period: measures change in mortality rate attributable to events in a time horizon, affecting all age and birth cohort groups.
3. Cohort: evaluates the effect on mortality rate explained by the person’s year of birth (or the generation he belongs), that is, a particular cohort of people has been exposed to specific risks or in its case to protective factors of any disease.

The model seeks to identify whether there are factors associated with women's age, their birth cohort and the period of death in the evolution of maternal mortality ratio for the period 2002–2014. It is worth mentioning that for estimating models, data from maternal deaths published by the Ministry of Health and INEGI was used, which is developed specifically for monitoring maternal mortality, with intentional search and full identification of the cause of death [14]. To estimate the results, the STATA statistical software was used.

## Results

### Maternal mortality: Situation in Mexico

Maternal deaths in Mexico present a higher incidence in metropolitan areas, 60% of these deaths occur in younger women between 20 and 34 years. More than 90% of deceased women had prenatal care for preventable causes associated with bad quality care. In recent years there has been a change in the main causes of maternal death, in previous years, the leading causes of maternal death corresponded to the pregnancy hypertensive disease, hemorrhage, puerperal infection, abortion and other causes. Currently, there has been an increase in the proportion of indirect obstetric causes which are not directly related to late access or deficiencies in the care quality [15].

The groups having more risks and with higher indicators in the maternal mortality ratio (MMR) are located in populations with social lags, so it is necessary to implement new strategies for preventing teen pregnancy and delaying active sexual life, as well as in patients older than 35 years to encourage these women to prevent concomitant illnesses. Due to the increase in indirect maternal deaths. Many of the maternal deaths occur during the postpartum period for which it is necessary to promote postpartum care from the pregnancy control [16].

**Fig 1:**
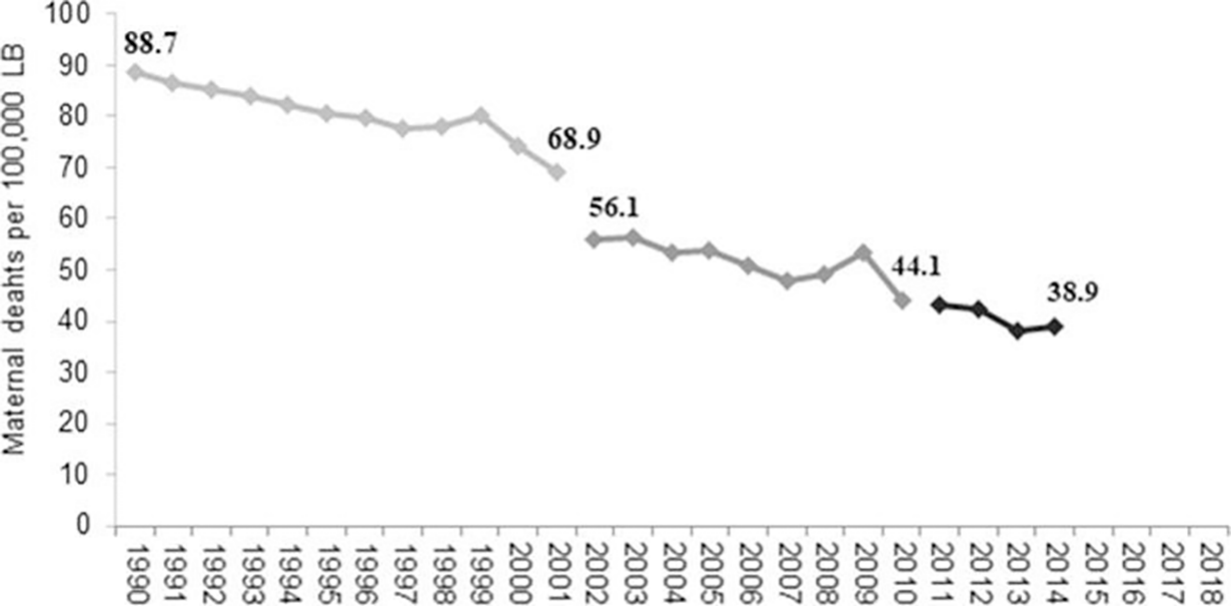
MMR evolution in Mexico, 1990–2014. LB: Live Births. Source: Dirección General de Información en Salud. Ministry of Health 2013.

The probability of dying due to maternal causes can be differentiated depending on the geographic location, social conditions and women's age. According to information from the Ministry of Health, those States that do register the worst indicators of marginalization, poverty and human development, also present the highest ratios of maternal mortality (Chiapas, Oaxaca and Guerrero). Mexico made the commitment of reducing maternal mortality 75% between 1990 and 2015 as part of the MDGs. From 1990 to 2014 the MMR per each 100,00 live births has decreased 56%. With the results achieved so far, it is estimated that in order to reach the goal, it will be necessary not to exceed 429 maternal deaths to achieve a maximum of 22.3 maternal deaths per 100,000 live births (Fig 1).

It is a major challenge to reach this figure since although maternal mortality has declined, if the average decline rate is kept during the period 2006–2013 (3%) the target set for 2015 until the year 2030 would be achieved.

As reflected in CONEVAL reports, poverty and inequality prevail in Mexico. In 2014, 46.2% of the Mexican population was in poverty and 9.5% in extreme poverty, states such as Chiapas, Estado de Mexico, Veracruz, Guerrero, Oaxaca and Puebla represent around 60% of the population in poverty and extreme poverty [8]. There is a direct correlation between MMR and poverty indicators. In 2014, the 100 municipalities with lowest human development index (HDI) had an MMR around 4 times the national average, and the 50 municipalities with the highest HDI only represent about 0.8 times the MMR observed in the national average (38.94). Of the women who died in the 100 municipalities with the lowest HDI 30.3% had no schooling whatsoever, 54.5% were under 30 years old and only 12% had no health insurance [17].

Of the total number of maternal deaths in 2014, 27% corresponds to women with high or very high degree of marginalization. Only 8.5% did not receive medical attention during childbirth (The proportion has reduced compared to 2002 that represented 13.8%. Even though in the 25 municipalities with less HDI represent 24%. In the 50 municipalities with higher HDI this proportion is 4%) and among the leading causes of death are: indirect obstetric causes (32%), hypertensive disease (21%), other pregnancy and childbirth complications (15%); and bleeding during childbirth and the puerperium (14%). All together represent 82% of maternal death causes [18].

The maternal mortality in Mexico presents two major trends, the highest frequency of maternal death cases occurs among women of low income who are residents of suburban or urban populations and in those rural, indigenous, and poor communities in remote areas where women do not have geographic, economic and social access to emergency obstetric services with resolution capacity. This group of deaths are concentrated in states with more marginalization; pregnant women living in these environments present a risk of dying two or three times higher than those that reside in the municipalities and states with higher HDI.

**Fig 2:**
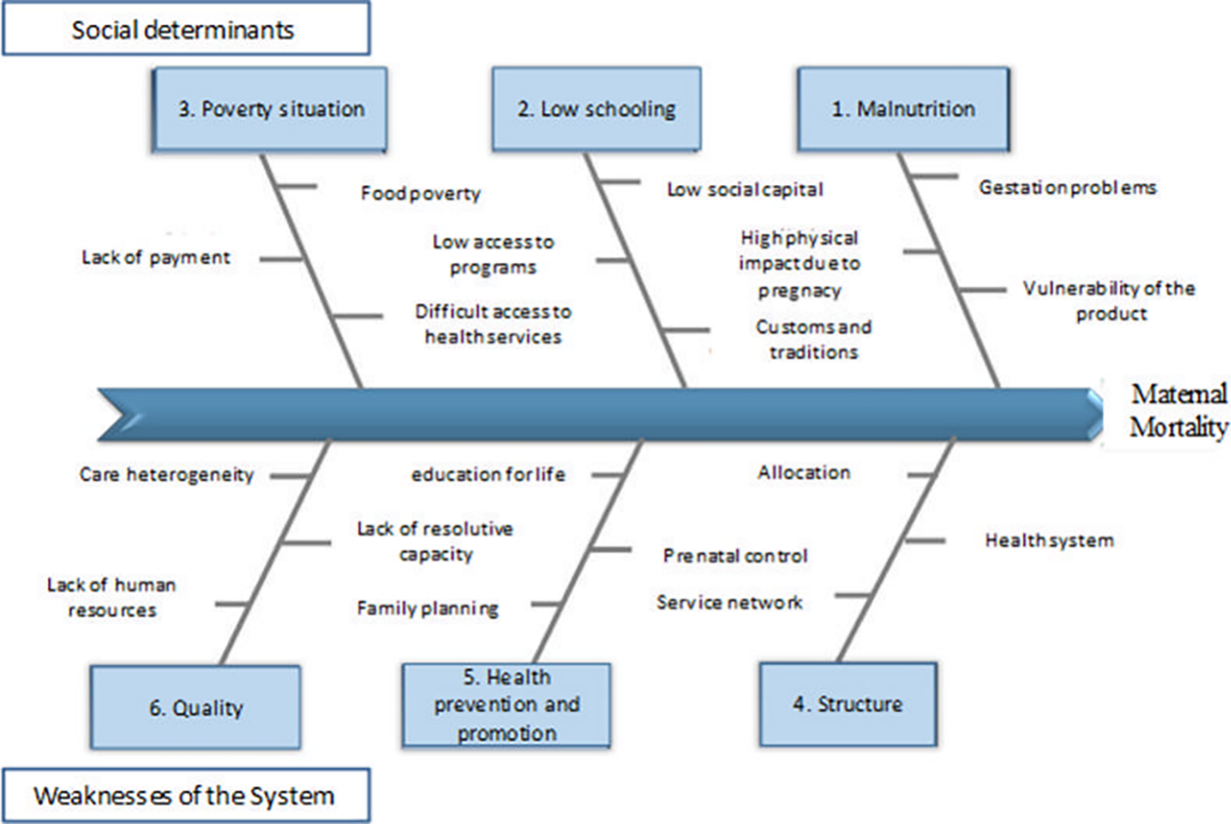
Causes of mother and child mortality in Mexico. Source: Own elaboration.

The causes of maternal death can be grouped according to the scope of action of the Health System and the population's social determinants. If referring to social determinants they can be: poverty, low education and poor nutrition, which are the main causes that go beyond the scope of the Health System. If causes inherent in the system are considered, aspects such as these would be highlighted: low coverage of family planning, poor care quality and weak and disjointed care networks (Fig 2).

If we compare Mexico with other countries in terms of maternal mortality, there are not many surprises. Mexico compared to other Latin American countries, in general, shows a more robust health system, therefore, better health indicators, still for the vulnerable population. In comparison with Latin America, in indicators such as fertility rate, use of contraceptives, place of delivery and type of received care, Mexico presents better indicators than the countries of Latin America, except for Brazil and Chile [18,19].

In contrast to developed countries, Mexico is really below the general indicators of maternal health. Mexico is one of the OECD countries with the highest rate of maternal mortality, in case of the prevalence in the use of contraceptives, Mexico is one of the countries with the lowest prevalence rates (Table 1). Which represents a great challenge in terms of prevention and promotion of maternal health.

**Table 1:** Maternal health, international comparison, 2015.

Country	Maternal Mortality. (100,000 live births)	Prevalence in the use of contraceptives% /1
**Spain**	5.0	65.7
**Germany**	6.0	70.1
**France**	8.0	76.6
**United Kingdom**	9.0	84.0
**Canada**	7.0	74.0
**United States**	14.0	78.6
**Chile**	22.0	64.2
**Costa Rica**	25.0	80.0
**Mexico**	38.0	70.9
**Brazil**	44.0	80.3
**Argentina**	52.0	65.3

Source: World Health Organization, (2015). /1% of women between 15–49 years that use come type of contraceptive, 2015 or the last available data.

In accordance with the main intergovernmental agencies such as UNFPA, UNICEF and WHO, and civil society organizations dedicated to reducing maternal mortality, there are five cost-effectivess interventions that directly attack this problem through the intervention of the Health System [20]:

1. Family planning.
2. Health Education.
3. Delivery care by professional staff.
4. Timely access to emergency obstetric care.
5. Access to safe abortion.

Likewise, there are demographic determinants that contribute indirectly to solve the problem, such as an increase in education levels, vulnerable groups having access to services or improvement in access to remote communities. In reference to the clinical aspects, it is necessary to ensure the adequate childbirth care by professionals based on the model of the three delays in obtaining emergency obstetric care, as well as the follow-up to the woman postpartum. The first delay, happens when the woman and her family do not recognize the symptoms of obstetric emergency and do not decide to promptly seek medical attention. In the second delay, the woman and her family make the decision, however this decision is not successful and go to services that do not have the capacity to provide primary care of the obstetric emergency or do not have capacity. The third delay occurs when the ability and the capacity of the health units is limited or did not offer quality care.

In reference to the most vulnerable groups of society, it is essential to improve the timely access to health services for women living in remote rural areas and strengthen social capital through health promotion and prevention, as well as generating social support networks.

The maternal mortality problem in Mexico represents a lag in the country's development, which is evident when compared to developed countries. Several measures have been implemented in order to resolve this problem, that until now have improved maternal mortality indicators but yet it is not enough, especially when it comes to the most vulnerable population. The structural characteristics of mexican health system represent a major institutional barrier to be able to achieve the set objectives, characteristic elements such as the system fragmentation, heterogeneity in quality and delivery of services between institutions, as well as the differentials in flow of resources between entities and institutions will be the big challenges to overcome in short and medium term. Any policy proposal to resolve the maternal mortality problem must consider these aspects, in addition to being consistent with the vision of the health system for Mexico, an integrated system with equitable access to quality health services.

#### Implemented plans and programs in Mexico

In Mexico, as in other developing countries, maternal health has become one of the strategic lines of the social welfare of the population. A set of programs have been implemented to reduce maternal mortality; in this section, plans and programs of greater relevance than have been implemented in Mexico in recent years are presented.

In recent years, the fight against poverty has emerged as one of the main objectives of the National Development Plans. The program which is more closely linked to issues raised on maternal mortality, is *PROSPERA* (for its acronym in Spanish) a Social Inclusion Program (before being *PROGRESA*). *PROSPERA* incorporates families, having the mother as head, which is intended to empower women. Additionally, three out of four of its strategies are focused on maternal care through a basic health package, self-health care through health education and strengthening the offer of health services. One of the key strategies is co-responsibility, which consists in the beneficiary families must register at the nearest health clinic, comply with recurring appointments and attend health education talks. Currently, *PROSPERA* benefits 6.1 million families, is working in nearly 115,000 towns, 2,456 municipalities and benefiting more than 300,000 infants and pregnant women, perceiving a budget of about $46 billion pesos via Branch 20 and $6 billion pesos through Branch 12 [21].

In the National Health Program 2000–2006 one of its main axes was the *Programa Arranque Parejo en la Vida* (APV for its acronym in Spanish), which places the problem of maternal mortality as a matter of inequality among Mexican women, in particular due to inequity in access to health services. The APV program supports its design in the Official Norm NOM-007-SSA2-1993 which establishes the criteria to meet and monitor women's health during pregnancy, childbirth and puerperium, as well as care of the newborn. Nevertheless, it is considered appropriate that the network of services operates from managing financial and material resources external to health sector institutions, which was one of the main problems that the program had to deal with, since it did not have additional resources to ensure the proposed service network in its scheme of operation [22].

The APV program was consolidated at the beginning of the implementation of the *Seguro Popular de Salud* (SP for its acronym in Spanish), a trans-sexennial initiative which entered in force on January 1^st^, 2004 and that it was proposed to give coverage to Mexicans who did not have social security, a group which accounted for approximately half of the population. Out of the 287 interventions covering nowadays *Seguro Popular*, 20 correspond to maternal and child care. Currently, *Seguro Popular* has 54.9 million affiliates and a budget of more than 75 billion pesos [23].

In the presidential administrations 2006–2012 and 2013–2018, the view from the federal scope was modified, it is recognized that maternal mortality is a process of inequality among women with different social position [16,24]. Mexico was committed, as part of the Millennium Development Goals (MDGs), to reduce maternal mortality by three quarters between 1990 and 2015, which means that for 2015 the MMR would have decreased to 22 maternal deaths per 100,000 live births. From 2009, strategies were targeted on eight states and focused on eliminating so called " three delays in obstetric care". The federal government, through the *Centro Nacional de Equidad de Género y Salud Reproductiva* (CNEGySR, for its acronym in Spanish) and on the basis of the APV program, launched various strategies to reduce maternal mortality, among which are:

- Healthy Pregnancy: consists in affiliating as priority all those pregnant women and their families to *Seguro Popular*. Currently, the program services 1.9 million pregnant women [23].
- *Seguro Médico Siglo XXI* (before *Seguro Médico para una Nueva Generación*): provides preventive care for the early detection of diseases for children born from December 1^st^, 2006 and on, who do not have social health protection, at the moment they have 5.5 million children under 5 years old [23].
- Program of Family Planning and Contraception: family planning and contraception is one of the most cost-effective interventions to reduce maternal and infant mortality; although it is defined as a strategic program and priority in the Programa Sectorial de Salud (Health Sector Program), yet there are no favorable impact results [25].
- General Collaboration for Obstetric Emergencies Care Agreement: as a support strategy of the fight against maternal mortality, in 2009 the Inter-Institutional Agreement between SSA, ISSSTE and IMSS through which every woman that present an obstetric care emergency should be addressed in any medical unit of the above-mentioned institutions. Even when the agreement was signed in 2009, this strategy had to overcome many institutional complications, to finally enter in force in August of 2011. In this scheme, from June 1st 2011 to September 25th 2015, there have been 3.792 maternal attendance [26,27].
- Comprehensive strategy to accelerate the reduction of maternal in Mexico: implemented in 2011, which applies that maternal mortality can be reduced by 40% to 2012 (based on 2006 figures) if overcoming factors that condition the three delays in accordance with the model adopted by the World Health Organization. Nonetheless, there is no available information about the results of such strategy [28].
- Specific Maternal and Perinatal Action Program 2013–2018: which aims to obtain results that impact maternal and perinatal health. Raises the need to improve the quality of health services, their effectiveness, monitoring and accountability; and in order to reduce the number of lags in health that affect the population. Reducing maternal and perinatal morbi-mortality, focusing on interculturality, giving priority to groups of high marginalization and in risk [29]. To date, there is no available information about the results of such program.

### Age-period-cohort effects in maternal mortality

A topic of broad interest to be analyzed is whether maternal mortality is attributable to social determinants or variables attributable to the health system. To analyze this point, a series of hypothesis tests are raised to compare samples of the population of maternal deaths according to their educational level, level of marginalization, condition of health affiliation and if the mother received medical attention or not during the delivery. When comparing the observed average deaths in the period 2002–2014 through a mean difference interesting results are found, because contrary to what was expected it is noted that there are no statistically significant differences between the average deaths of mothers with and without health affiliation due mainly to the expansion of coverage driven in recent years. For their part, the educational and marginalization levels, and receiving or not medical care during delivery did show differences in average deaths (Table 2). According to what was expected, the influence of associated variables with the social conditions and to the health system that impact maternal mortality is identified.

**Table 2:** Mean difference: Social determinants and the health system.

Social determinants	Average	Standard error	Confidence interval to 95%	(t)	p-value* (significance 5%) H0: Diference = 0 H1: Diference≠0 H1b: Diference>0 H1c:Diference<0
**Uneducated**	27.69	0.079	(27.53,27.85)		
**Professional**	8.00	0.274	(7.46, 8.54)		
**Difference**	2.008	0.497	(1.029,2.988)	4.0399	Pr(|T| > |t|) = 0.0001 Pr(T > t) = 0.0000 Pr(T < t) = 1.0000
**High and very high marginalization level**	30.68	0.649	(29.40,31.97)		
**Low and very low marginalization level**	54.84	1.000	(52.86,56.82)		
**Difference**	-24.158	1.192	(-26.506, -21.810)	-20.266	Pr(|T| > |t|) = 0.0000 Pr(T > t) = 1.0000 Pr(T < t) = 0.0000
**Attributable to the Health System**					
**Without medical care**	10.43	0.375	(9.69, 11.18)		
**With medical care**	83.33	1.274	(80.81, 85.86)		
**Difference**	-72.9	1.32795	(-75.516, -70.283)	-54.896	Pr(|T| > |t|) = 0.0000 Pr(T > t) = 1.0000 Pr(T < t) = 0.0000
**Without health affiliation**	48.73	2.030	(44.71,52.75)		
**With health affiliation**	48.38	1.506	(45.40,51.36)		
**Difference**	0.350	2.527327	(-4.628, 5.328)	0.1385	Pr(|T| > |t|) = 0.8900 Pr(T > t) = 0.4450 Pr(T < t) = 0.5550

*Unpaired samples are taken and the variance of the samples is the same.

Source: Own estimations based on information from the Ministry of Health. Mexico, 2002–2014.

In case of the selected variables as social determinants like the level of schooling, the null hypothesis that there is no difference between the average deaths in women with no schooling and in women with vocational education is rejected. There is enough statistical evidence to say that there is no difference and that the average number of deaths in women with no schooling is higher than those with vocational education. In reference to the level of marginalization the null hypothesis that there is no difference between deaths of women in areas with a high and very high level of marginalization in relation to those of low and very low level of marginalization, if there is a difference between means but not in the expected way as the average of deaths among women in areas of low and very low marginalization is greater, this can be attributed to mobility of patients toward areas with the greatest health infrastructure and lower level of marginalization in cases where birth is complicated, and that culminates in death in a different area of residence.

From the group of variables attributable to the Health System, the difference between the average number of deaths of women who did not receive medical attention compared to those who did, it is noted that the null hypothesis is rejected. There is a difference between averages, but not in the expected way because when observing the result of the second test the alternative hypothesis (difference between averages is less than zero) is accepted, meaning that women's deaths with medical attention is higher than those who did not receive it. This is explained when women go late to receive health care, the level of women who do not go to receive delivery care is very low. Finally, the status of health affiliation shows that there are no statistically significant differences between averages of deaths of women with affiliation in health respecting the non-affiliated; the affiliation does not generate differences in the average of deaths.

It is impossible to separate the social determinants and the variable attributable to the Health System when considering policy options to solve the maternal mortality problem in Mexico. Care Quality and timeliness are essential aspects that must be considered in the development of a targeted public policy to reduce maternal mortality in vulnerable groups. Half of the pregnant women who die in the country does not arrive in a timely manner to receive hospital services. That is, they do not have social and cultural capital to access quality care. Among the main characteristics of women and their families who condition inequalities between the female gender [30] highlight:

- The economic capital: which includes the material resources the family has.
- The human capital: constituted by the education level and knowledge-information about the complications during maternity leave, and the level of speaking and understanding Spanish.
- The social capital or support networks: that allow to mobilize material and human resources that enable different options to solve problems.

Strengthening the access to health services and service quality, coupled with a good policy of social communication and health education can have an impact on empowering the human capital, helping to promote a culture of health and improving the social capital through support networks. The Mexican Health System presents singularities of great impact in all health topics, the incomplete decentralization in Mexico in the 90's, which culminated with the creation of the *Sistema de Protección Social en Salud* (SPSS for its acronym in Spanish. System of Social Protection in Health), generated a fragmented system with three main providers of public health services. Which matches the three different systems both financing and service provision. This is reflected in the access and quality services that the population receives.

These aspects of the Mexican health system are clearly reflected in the maternal mortality as the health institutions that receive more resources are not necessarily those who have more productivity in terms of maternal care, mainly due to different financing schemes of the institutions providing services. For example, *Seguro Popular*, which in 2013 received 14% of the total Health Sector budget and helped through medical units of the Ministry of Health to about 1,275 million hospital discharges related to maternal causes, almost more than two times the number of discharges per maternal causes that the IMSS attended (575,000 discharges), institution that received 45% of the budget [31]. There is inequality in the distribution of resources in the Mexican Health System due to its organic-functional structure attributable to diverse reforms they have been through. The disparity in terms of financing between the population with social security and those without has declined in recent years, but there is still a gap between institutions and between entities. The *Seguro Popular* has been one of the mechanisms that has helped to reduce this gap and significantly expand health coverage to people without social security.

The maternal mortality continues to be a challenge to overcome in Mexico, because even with efforts made since the implementation of policies, the indicators show that there are challenges to overcome, especially in the most vulnerable sectors that present indicators which are very distant from the national average.

The EPC model estimates are presented separately, to identify if there is really is an impact attributable to the age of the mother, death period and birth cohort in maternal mortality in Mexico. The results of the estimation of the APC model are shown in Table 3, it is observed that all three effects are statistically significant at 95% confidence, but not steadily in the different cuts performed on the variables.

**Table 3:** Age-period-cohort model for MMR.

D	Coef.	OIM Std. Err.	Z	P>|Z|	[95% Conf. Interval]	
**_spA1_intct**	-6.7092	0.0341	-196.6300	0.0000*	-6.7760	-6.6423
**_spA2**	-0.0704	0.0468	-1.5000	0.1320	-0.1623	0.0213
**_spA3**	-0.2662	0.0370	-7.1800	0.0000*	-0.3389	-0.1935
**_spA4**	0.2911	0.0257	11.2900	0.0000*	0.2405	0.3417
**_spA5**	0.1368	0.0215	6.3500	0.0000*	0.0946	0.1791
**_spA6**	0.0792	0.0182	4.3500	0.0000*	0.4359	0.1149
**_spP1**	0.0004	0.0086	0.0500	0.9600	-0.0165	0.0174
**_spP2**	0.0098	0.0084	1.1700	0.2430	-0.0066	0.0263
**_spP3**	-0.0175	0.0087	-2.0000	0.0450*	-0.0347	-0.0003
**_spP4**	-0.0065	0.0081	-0.8000	0.4250	-0.0226	0.0095
**_spC1_1drft**	-0.0304	0.0022	-13.2800	0.0000*	-0.0349	-0.0259
**_spC2**	0.0343	0.0313	1.1000	0.2720	-0.0270	0.0957
**_spC3**	0.0128	0.0238	0.5400	0.5890	-0.0338	0.0595
**_spC4**	0.0275	0.0131	2.1100	0.0350*	0.0019	0.0532
**_spC5**	0.0220	0.0118	0.1900	0.8490	-0.0209	0.0254
**Number of obs = 506**						
**Log likelihood = -144.088784**						
**AIC = 5.648572**						
**BIC = -2476.342**						

Source: Own elaboration based on information from the Ministry of Health and INEGI.

*Statistically significant at 95% confidence.

**Fig 3:**
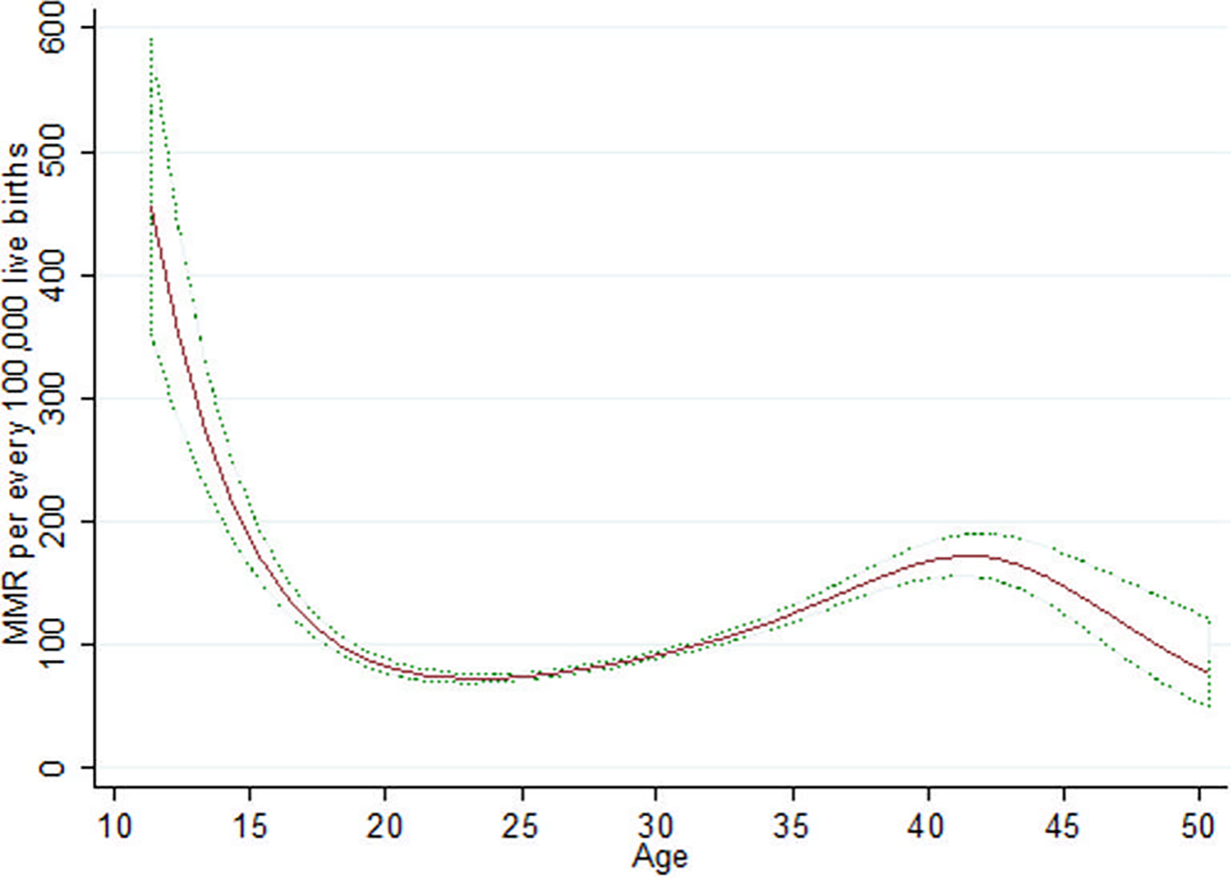
Age effect in maternal mortality. Source: Own elaboration based on information from the Ministry of Health and INEGI.

Fig 3 shows the evolution of Maternal Mortality Ratio per evert 100,000 live births respecting the age of the mother. It is noted that maternal deaths are higher at early and advanced ages, this is explained by clinical reasons due to physical wear when woman are giving birth to a child, the risks of death are greater for women under 15 and over 35 years old.

The continuous line represents the MMR per age and the dotted lines their respective confidence intervals. This result is consistent with the analyzed problems, due to teenage pregnancies generate greater risks for the mother. The age range of 35 to 45 years represents another risk segment for the mother, which declines according to increasing age, this for biological reasons that limit the likelihood of getting pregnant for women older than 50 years. The fact that there is greater mortality in young women implies that the health system should be more aware about the problem of teenage pregnancy through better prevention and promotion of reproductive health.

**Fig 4:**
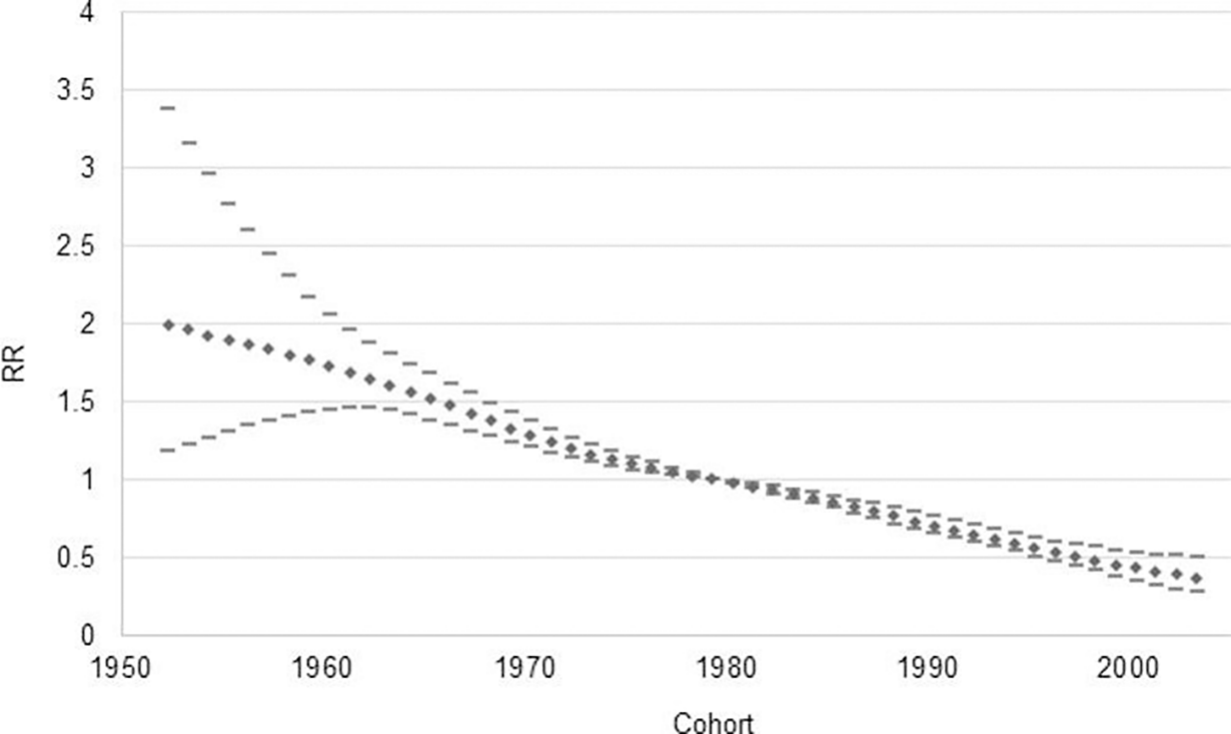
Cohort effect in MMR. Source: Own elaboration based on information from the Ministry of Health and INEGI.

The cohort effect for its part shows significant results in terms of the positive evolution of maternal health in Mexico and social determinants. There is a clear downward trend, which shows that for the cohorts of women born in recent years the probability of death is lower in terms of relative death risk in relation to the reference cohort (1980) (Fig 4).

This result shows that actions implemented in terms of social development and the increase in access and quality of health services, have decreased death risk for younger generations. Women born before 1980 have more death risk when being mothers, than women belonging to further cohorts. Another important factor of this result can be the reduction of global fecundity rate in Mexico, which has decreased in last years, going from an average of 4.8 children per women in 1980 to 2.2 in 2014 [32].

**Fig 5:**
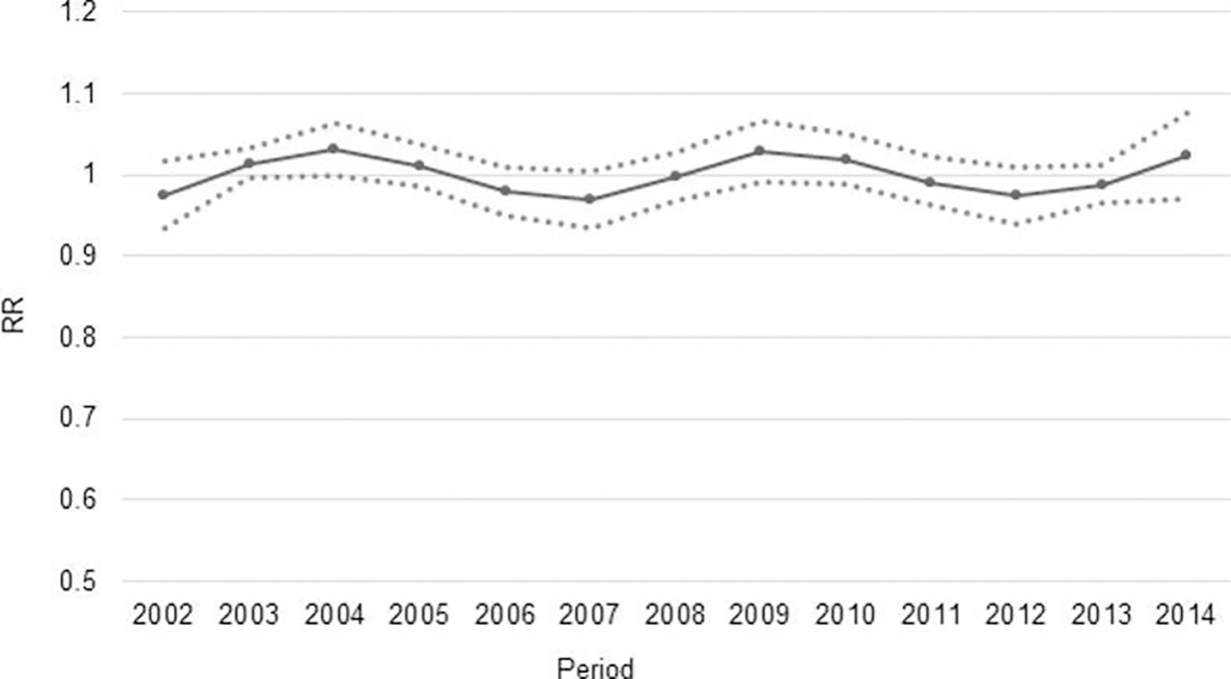
Period effect in MMR. Source: Own elaboration based on information from the Ministry of Health and INEGI.

The last analyzed effect is the period effect, which seeks to capture the effect of an event in the time that it generated significant implications for maternal mortality. Either a policy or innovative treatment that have helped to decrease mortality. Fig 5 shows the period effect for MMR in Mexico.

The period effect does not show a clear trend, there are fluctuations in time, but it is not possible to identify a dramatic effect of any event in a year that determine a significant increase or decrease in relative risk of maternal death in function of the period.

While series of policies and programs implemented should be reflected in the model by using the effect period, it is clear that in health policies, the result is not measurable in short term, it is worth mentioning the fluctuations in the period effect in intervals near to the beginning and end of each six-year term. It is likely that there is an effect attributable to the budget cycle in the implementation of plans and programs, which generates impacts as exercised in the budget and the continuity of certain programs at the beginning of each administration is defined. It is necessary to be cautious with these results because the fluctuations having the relative risk in the MMR regarding the period effect may be multifactorial.

## Discussion

The problem of maternal mortality has been an issue of the government agenda for some years now; it is a problem of national interest since it is a reflection of inequality and social justice in a society. The goal of reducing maternal mortality still represents a challenge since the results obtained to date are positive but not sufficient, and to greater extent if the results are compared at national level regarding to what has been observed in the 100 municipalities with the lowest HDI, where inequity and access barriers remains.

Comparing average deaths observed in the period 2002–2014 it is noted that there are no statistically significant differences between average deaths of mothers with and without health affiliation, this, in part explains the expansion of coverage driven in recent years. For its part, educational and marginalization levels, and not receiving medical care during childbirth did presented differences in average deaths.

Two major policy lines are highlighted for solving the problem. On one hand, actions in terms of the *PROSPERA* program, which is focused on the development of capacities and with a health component. However, it does not have sufficient coverage for vulnerable populations and the health component has no specific interventions for women. The *Seguro Popular* is in another line of action that seeks to generate broad impact in health, as one of its objectives is to give greater health services coverage for the population that does not have any affiliation, and thus, allow access to vulnerable population.

The *Seguro Popular* contemplates a defined group of interventions for beneficiary population; however, it does not specify any package of services for vulnerable populations. With the emergence of the *Seguro Médico Siglo XXI*, the objective was to break social barriers that because of ignorance prevented access to *Seguro Popular*, allowing children (and their families) who were born after the December 1^st^, 2006 to be incorporated to *Seguro Popular*. Subsequently, the program Healthy Pregnancy was created, which provides pregnancy care for pregnant women and seeks to ensure an uncomplicated birth and a healthy newborn. One of the major weaknesses of the specific health programs that have as axis *Seguro Popular* are those operating programs, due to the decentralized structure of the health system- It is necessary to enter into agreements with different government levels that many times depend on their political will. Most causes of maternal mortality can be attacked from social determinants and health promotion and prevention. For that, it is necessary to have a comprehensive focused program.

The APC model raised to evaluate the evolution of maternal mortality in Mexico shows that significant progress has been achieved in the improvement of social determinants and in the Mexican Health System. The model shows that birth cohorts previous to 1980 had more probability of maternal death than birth cohorts of further years. This implies that generations of recent mothers that have better birth conditions, both social and in terms of health. In terms of the ages of death, results displayed by the model are consistent, because it identifies a significant problem in mortality of women under 15 years old, the latter is widely associated with the problem of teenage pregnancy, a problem that should be addressed from the perspective of promotion and prevention of reproductive health. The period factor does not present a clear trend in terms of the effect of any policy when referring to risk of maternal death in time.

For the latter, it is important to highlight political actions implemented have had positive results when achieving an important reduction in the past years MMR. However, it is important to note the persistence of inequalities between regions in the country, keeping maternal mortality indicators at high levels nationally in those populations with the lowest HDI, which indicates that there is a need for greater targeting of programs.

### Recommendations

Derived from the analysis of alternatives of implemented policies that are currently in operation and of the identified main causes of maternal mortality, the following recommendations are proposed to improve and search for optimal alternatives to address the problem of maternal mortality in vulnerable populations in Mexico in a long-term vision (S2 Table).

- Strengthen prenatal care, because there are access and use of this service barriers.
- Regulate and strengthen the referral and counter-referral system, as well as mechanisms to ensure the effective Agreement implementation for the inter-agency emergency obstetric care.
- Ensure resolution capability of the medical units for pregnancy attention and its complications.
- Establish mechanisms that guarantee universal coverage of professional care during the delivery, preferably in hospital units.
- Training and updating health staff in terms of obstetrics.
- Strengthening and expanding contraceptive coverage in the country, with the objective to influence particularly in the occurrence of early pregnancies.
- Interventions of health promotion and education focused on early recognition of alert signs of obstetric complications and pregnancy care.
- The highest frequency of maternal deaths in women with no schooling and in poverty conditions highlights the urgent need to continue to reinforce cross-sectoral action.
- It is necessary to remove access barriers of health care, because among the major impediments to adequately comply with prenatal care and timely care of obstetric emergencies, availability of medical units, quality of communication channels, distance to the medical units and transfer cost are emphasized.
- Any proposed policy should be consistent with the vision of the Health System in Mexico, a universal and integrated system.
- A policy focused to reduce maternal mortality is seen as a viable alternative, the conjunction of the health component of *PROSPERA* program and *Seguro Popular* in a component focused to reduce maternal mortality in vulnerable populations.

## Supporting information

S1 Dataset Maternal mortality 2002–2014. (ZIP)

S1 Table Age-period-cohort model. (DOCX)

S2 Table SWOT matrix of the main implemented plans and programs in Mexico to attack maternal mortality. (DOCX)
