# Supplementary material for: Maternal mortality in Mexico, beyond millennial development objectives: An age-period-cohort model
Source: PLoS One. 2018 Mar 21;13(3):e0194607. doi: 10.1371/journal.pone.0194607 (PMC5862485; doi:10.1371/journal.pone.0194607)
Supplement: S1 Table — (DOCX) [file pone.0194607.s002.docx]

**S1. Table. Age-Period-Cohort model**

|  |
| --- |
| $\boldsymbol{log}\left\{ \frac{\boldsymbol{\theta}_{\boldsymbol{ijk}}}{\boldsymbol{N}_{\boldsymbol{ijk}}} \right\}\boldsymbol{=}\boldsymbol{\mu}\boldsymbol{+}\boldsymbol{\alpha}_{\boldsymbol{i}}\boldsymbol{+}\boldsymbol{\beta}_{\boldsymbol{j}}\boldsymbol{+}\boldsymbol{\gamma}_{\boldsymbol{k}}$ |
|  |
| **Where:** |
| $\boldsymbol{log}\left\{ \frac{\boldsymbol{\theta}_{\boldsymbol{ijk}}}{\boldsymbol{N}_{\boldsymbol{ijk}}} \right\}$, logarithm of the expected value of mortality rate or incidence |
| $\boldsymbol{\mu}$, represents the average effect |
| $\boldsymbol{\alpha}_{\boldsymbol{i}}$, represents the age effect |
| $\boldsymbol{\beta}_{\boldsymbol{j}}$, represents the period effect |
| $\boldsymbol{\gamma}_{\boldsymbol{k}}$, is the cohort variable |

Source: Own elaboration.
