# Supplementary material for: Maternal mortality in Mexico, beyond millennial development objectives: An age-period-cohort model
Source: PLoS One. 2018 Mar 21;13(3):e0194607. doi: 10.1371/journal.pone.0194607 (PMC5862485; doi:10.1371/journal.pone.0194607)
Supplement: S2 Table — (DOCX) [file pone.0194607.s003.docx]

**S2 Table. SWOT matrix of the main implemented plans and programs in Mexico to attack maternal mortality**

| Program | Strengths | Weaknesses | Opportunities | Threats |
| --- | --- | --- | --- | --- |
| *PROSPERA* | Coverage of a segment of the vulnerable population.  Health content of the program. | Insufficient coverage.  There is no focused health component to fight maternal and child mortality. | Is a high impact program.  Does not cover the total of vulnerable population if being beneficiary. | The budget sustainability is not guaranteed. |
| *Seguro Popular* | Provides health coverage to population without health insurance.  It is a tripartite scheme which seeks to ensure health access of the population. | It is a funding mechanism and does not have its own infrastructure to provide health services. | Redesign the health system.  It is a mechanism created to fight social inequality in health. | Budgetary sufficiency.  The fragmentation of the health system can generate that the *Seguro Popular* is yet another institution of the system and the Universal Health System is not accomplished. |
| Healthy Pregnancy | It is a program focused on the attention of pregnant women and newborns. | Is not reaching the most vulnerable population.  Their actions only contemplate affiliation of pregnant women to the *Seguro Popular*. | Program that can be enhanced at national level and is targeted to vulnerable population.  It is possible to add the education component to mother and child health. | Isolated effort, which only remains as a plan and does not operates. |
| *Seguro Médico Siglo XXI* | Protection of vulnerable groups. | Affiliation is voluntary and in most communities in extreme poverty there is no information of the program and in many cases do not have the required documentation for being part of the program. | In an integrated scheme the program to generate more impact on the population can be enhanced. | Sheltered and disintegrated national program policy.  Adverse selection. |

Source: Own elaboration based on *CONEVAL*, (2012). Consistency and results evaluation 2011-2012. *Seguro Médico para una Nueva Generación. Mexico: CONEVAL*. CONEVAL (2012). Strategic Evaluation of Maternal Mortality in Mexico 2010: *socio-*demographic characteristics of pregnant women that hinder their effective access to health institutions. Mexico: CONEVAL. *SEDESOL*, (2012). *Oportunidades*, 15 years of results. Mexico: SEDESOL.
